# Systematic bioinformatics analysis identifies shikonin as a novel mTOR pathway inhibitor in triple-negative breast cancer progression

**DOI:** 10.3389/fphar.2025.1603093

**Published:** 2025-08-29

**Authors:** Wenna Liu, Qingqing Liu, Jingyue Yao, Dan Wu, Wenxuan Li, Zhenkai Fu, Minggao Zhao, Ying Liang

**Affiliations:** ^1^ Precision Pharmacy and Drug Development Center, Department of Pharmacy, Tangdu Hospital, The Fourth Military Medical University, Xi’an, Shaanxi, China; ^2^ Laboratory of RNA Epigenetics, Institutes of Biomedical Sciences, Shanghai Medical College, Fudan University, Shanghai, China; ^3^ School of Basic Medical Sciences, Peking University, Beijing, China

**Keywords:** triple-negative breast cancer, shikonin, molecular docking, bioinformatic analysis, MTOR signaling

## Abstract

Triple-negative breast cancer (TNBC) remains a therapeutic challenge due to its resistance to conventional therapies and poor prognosis. Shikonin, a natural compound derived from Lithospermum erythrorhizon, has demonstrated antitumor potential in TNBC, though its molecular mechanisms remain unclear. In this study, shikonin’s antitumor effects were systematically evaluated using colony formation, wound-healing assays, transcriptomic profiling, and molecular docking. Results demonstrated that shikonin markedly inhibited TNBC cell proliferation and migration. Transcriptomic analysis identified downregulation of key mTOR signaling pathway genes (MTOR, CCND1, CDK6) post-treatment. Molecular docking confirmed direct binding between shikonin and the mTOR protein, suggesting mTOR pathway inhibition as a critical mechanism. Of note, the PI3K/AKT/mTOR axis is frequently hyperactivated in TNBC to regulate tumor proliferation and survival, yet existing mTOR inhibitors show limited efficacy in this subtype due to feedback activation of compensatory pathways and off - target effects that reduce their specificity for TNBC. Our findings highlight shikonin’s ability to target mTOR-related signaling, offering a novel strategy for TNBC treatment. This study provides foundational insights into shikonin’s molecular action, emphasizing its potential as a natural mTOR inhibitor tailored for TNBC. Further exploration of shikonin’s therapeutic applications could address the urgent need for targeted therapies against this aggressive breast cancer subtype, bridging gaps in current clinical approaches.

## 1 Introduction

Breast cancer is a complex and heterogeneous disease comprising four primary molecular subtypes: luminal A, luminal B, human epidermal growth factor receptor 2 (HER2)-positive, and triple-negative breast cancer (TNBC). Notably, TNBC is uniquely characterized by distinct biological traits and clinical behavior, making it the most difficult to treat and associated with the poorest prognosis (Derakhshan and Reis-Filho, 2022; Li Y. et al., 2022). TNBC is specifically defined by the absence of estrogen receptor (ER), progesterone receptor (PR), and HER-2, accounting for approximately 15% of all breast cancer cases. Its distinct pathological features, including high proliferative activity, significant metastatic potential, and aggressiveness, render current standard breast cancer treatments less effective than those for other TNBC subtypes. Given its high recurrence rate and poor prognosis, the development of novel therapeutic strategies specifically targeting TNBC is urgently required (Bi et al., 2022).

Shikonin, an active ingredient extracted from the roots of the plant *Lithospermum erythrorhizon*, has shown tremendous potential for cancer treatment, especially TNBC, in recent years (Guo et al., 2019). It has been reported that shikonin induces ferroptosis in osteosarcoma by indirectly regulating the HIF-1α/HO-1 axis, thereby inhibiting tumor progression (Lu et al., 2024). In addition, shikonin can exhibit anti-cancer activity against non-small cell cancer cells that are resistant to paclitaxel by inhibiting the transmission of NEAT1 and AKT signals (Zang et al., 2020). In particular, there are some studies on the inhibitory effect of shikonin on the progression of TNBC. Specifically, shikonin inhibits epithelial–mesenchymal transition (EMT) by suppressing the miR-17-5p/PTEN/Akt pathway, thereby reducing the migration and invasion of TNBC cells (Bao et al., 2021). Additionally, shikonin promotes the self-ubiquitination and degradation of cIAP1 and cIAP2 to induce a decrease in the ubiquitination of RIP1, thereby inhibiting the activation of the pro-survival signaling pathway and accelerating the necrosis of MDA-MB-231 cells (Liang et al., 2021). However, the specific molecular mechanisms underlying the action of shikonin in TNBC remain unclear. Therefore, further in-depth research on the mechanism of action of shikonin is of great significance for the development of new treatment strategies for TNBC.

It is widely recognized that mutations in proto-oncogenes play a pivotal role in the pathogenesis of tumors (Martinez-Jimenez et al., 2020). Among these, abnormal activation of the proto-oncogene mTOR has emerged as a hallmark of tumorigenesis (Hua et al., 2019). In breast cancer, the PI3K/AKT/mTOR signaling pathway is a critical signaling cascade that is highly susceptible to hyperactivation. This aberrant hyperactivity is closely associated with the excessive proliferation and uncontrolled growth of tumor cells (Miller et al., 2011; Ero et al., 2012). mTOR serves as a central regulator in this pathway, controlling downstream processes intricately linked to cell proliferation, growth, development, and mRNA transcription (Hua et al., 2019; Soulard and Hall, 2007). Notably, downstream target genes regulated by *MTOR*, such as *CCND1* and *CDK4/6*, exhibited a strong correlation with expression levels, cell cycle regulation, and cell proliferation status. Inhibiting the activity of these targets can effectively arrest cell cycle progression, thereby suppressing the proliferation and growth of tumor cells. Multiple studies have consistently confirmed that inhibiting the expression of mTOR and its downstream target genes has a pronounced effect on halting the malignant progression of breast cancer (Soulard and Hall, 2007; Goel et al., 2022). Although mTOR inhibitors for breast cancer treatment are currently at various stages of development, their therapeutic efficacy in TNBC remains significantly lower than in other types (Li Y. et al., 2022; Xu et al., 2021). This is mainly due to the unique biological characteristics of TNBC, which can potentially trigger the feedback activation of compensatory signaling routes and induce off - target impacts when existing mTOR inhibitors are administered, thereby diminishing their specificity and therapeutic efficacy (Browne et al., 2024; Gough et al., 2024). Therefore, the development of effective mTOR inhibitors, specifically tailored for TNBC, has immense clinical value and therapeutic significance.

Recent advancements in research on natural compounds have provided evidence that certain natural medications can effectively hinder tumor progression by inhibiting mTOR signaling (Wu et al., 2021; Ganesan et al., 2024). Among these, shikonin has been reported in various tumor models to exert antitumor effects by modulating the mTOR pathway (Ni et al., 2018; Li J. et al., 2022). However, whether shikonin influences the occurrence and development of TNBC by regulating mTOR - related signaling pathways remains unclear, and the underlying mechanisms require further in - depth investigation. Given the urgent need for effective TNBC treatments and the potential of mTOR - targeted natural compounds, exploring shikonin’s role in TNBC could provide novel insights to fill the existing treatment gap.

In this study, we aimed to investigate the potential of shikonin as a therapeutic agent for TNBC. Given the urgent need for effective treatments for this aggressive cancer subtype, we hypothesized that shikonin could inhibit the malignant progression of TNBC by targeting specific molecular pathways. To test this hypothesis, our approach encompassed a combination of functional assays, transcriptomics, and molecular docking. Initially, we planned to use functional assays such as colony formation and wound - healing assays to obtain preliminary evidence of shikonin’s inhibitory effect on TNBC cells. Subsequently, we intended to employ mRNA - seq high - throughput sequencing technology to analyze changes in gene expression profiles before and after shikonin treatment. By conducting Kyoto Encyclopedia of Genes and Genomes (KEGG) enrichment analysis on the sequencing data, we aimed to identify key signaling pathways involved in shikonin’s action. Finally, we planned to validate the direct interaction between shikonin and target proteins using molecular docking techniques. Through this multi - faceted approach, we sought to elucidate the molecular mechanism underlying shikonin’s antitumor effects in TNBC.

## 2 Methods and materials

### 2.1 Cells and reagents

The cell lines used in our study were the human breast cancer cell lines MDA-MB-231 and MDA-MB-468, both purchased from the Cell Bank of the Chinese Academy of Sciences (Shanghai, China). MDA-MB-231 cells were cultured in Dulbecco’s Modified Eagle’s Medium (DMEM, Gibco™, C11995500BT) supplemented with 10% fetal bovine serum (FBS, Excell Biotech, 12B211, Jiangsu, China) and 1% penicillin/streptomycin (Sangon Biotech, E607011, Shanghai, China), while MDA-MB-468 cells were maintained in Roswell Park Memorial Institute (RPMI) 1640 medium (Cytiva, AJ30752991, Shanghai, China) containing 10% FBS (Excell Biotech, 12B211, Jiangsu, China) and 1% penicillin/streptomycin. The cells were cultured under conditions of 5% CO_2_ at 37 °C. Phosphate Buffered Saline (PBS, Sangon Biotech, E607008, Shanghai, China). Trypsin (Sangon Biotech, E607002, Shanghai, China). The detailed information of all reagents is listed in Table 1 as below. All cell lines were authenticated by short - tandem repeat (STR) profiling to confirm their identity. All cell lines were regularly tested for *mycoplasma* contamination using a commercially available *mycoplasma* detection kit (MedChemExpress, HY-K0552-100, Shanghai). Only *mycoplasma* - free cell lines were used for the subsequent experiments.

**TABLE 1:** Reagent information.

Reagent	Producer	Catalogue number
DMEM	Gibco™	C11995500BT
FBS	Excell Biotech	12B211
penicillin/streptomycin	Sangon Biotech	E607011
RPMI 1640 medium	Cytiva	AJ30752991
PBS	Sangon Biotech	E607008
Trypsin	Sangon Biotech	E607002
*Mycoplasma* detection kit	MedChemExpress	HY-K0552-100
Shikonin	Meilunbio	MB7082
CCK8 kit	Enogene	E1CK- 000208
Trizol	Sangon Biotech	B511311
Reverse transcription kit	TaKaRa Biotech	RR047A
FastStart Essential DNA Green Master Mix	Roche	6924204001

Shikonin (Meilunbio, MB7082; purity >98%, China) was prepared as a 50 μM stock solution in DMSO and stored at −20 °C in the dark. When needed, it was diluted to the desired concentration with the medium used for culturing the cells.

### 2.2 IC_50_ calculation

MDA-MB-231 (8,000 per well) and MDA-MB-468 (15,000 per well) cells were cultured in a 96-well plate. After 24 h of incubation, shikonin was added at various concentrations (for MDA-MB-231: 0, 0.125, 0.25, 0.5, 1.0, 2.0, 4.0 μM; for MDA-MB-468: 0, 0.16, 0.32, 0.64, 1.28, 2.56, 5.12 μM). The concentration gradients were determined based on the results of the preliminary experiments. After 24 h of exposure to shikonin, medium containing 10% CCK8 reagent was added, and the cells were incubated for 1 h. During the CCK8 experiment, we first removed the culture medium from the 96-well plate, then mixed CCK8 with DMEM, and finally added 200 μL of the 10% (v/v) CCK8 solution to the 96-well plate. The absorbance was measured using a microplate reader (Infinite M200 PRO, TECAN, Switzerland) at 450 nm. Subsequently, the IC_50_ (half maximal inhibitory concentration) value of shikonin was calculated. IC_50_ values by plotting a concentration - response curve using non - linear regression with the four - parameter logistic equation. The drug concentrations used in the subsequent experiments in the article were all determined based on the IC_50_ values obtained from the CCK8 experiment.

Inhibition ratio: [(Ac-As)/(Ac-Ab)]x100%. Ab: Absorbance of the blank group; Ac: Absorbance of the control group; As: Absorbance of the sample.

### 2.3 Cell proliferation assay

In a 96-well plate, MDA-MB-231 (2,000 per well) and MDA-MB-468 cells (4,000 per well) were incubated for 24 h. Subsequently, shikonin was added to the TNBC cells at specific concentrations (MDA-MB-231: 0, 0.484 μM; MDA-MB-468: 0, 1.070 μM). Cells were further incubated for 5 d, and cell viability was measured every 24 h using a Cell Counting Kit-8 (CCK8 kit, Enogene, E1CK- 000208, Jiangsu, China).

### 2.4 Colony formation assay

In a six-well plate, 2,000 MDA-MB-231 and MDA-MB-468 cells were seeded per well. After 24 h of incubation, shikonin was added at specified concentrations (0, 0.484, 0.968 μM for MDA-MB-231; 0, 1.070, 2.140 μM for MDA-MB-468). After an additional 24 h of incubation, the medium containing shikonin was removed and the cells were cultured for 14 days with standard growth medium as above mentioned in Cells and Reagends section. Visible cell colonies were formed, at which point the culture medium was discarded, and the wells were washed once with PBS. The colonies were then stained with crystal violet containing 2% paraformaldehyde for 15 min, after which the crystal violet was aspirated, and the wells were washed twice with PBS. The plate was allowed to air-dry before images (Images were captured using a standard mobile camera under consistent lighting conditions for all groups) were captured. Finally, the number of colonies was counted, and the cell colony formation rate was calculated. ImageJ was used to analyze the images and count the number of colonies formed in each group of 6-well plates.

Coloning efficiency (%) = the number of colonies/the number of cells inoculated. When using ImageJ for analysis, all the parameter settings of the images were kept consistent to minimize the errors caused during the data analysis process.

### 2.5 Wound-healing assay

MDA-MB-231 and MDA-MB-468 TNBC cells were seeded in six-well plates at a density of 0.8 × 10^5^ and 1.5 × 10^5^ cells/well, respectively. When the cell density reached 70%–80%, a straight line was scratched in the middle of the cells using a 10 μL pipette tip, and the cells were incubated with medium containing shikonin for 24 h at different concentrations (MDA-MB-231: 0, 0.484, and 0.968 μM, MDA-MB-468: 0, 1.070, and 2.140 μM). Photographs (Cytation™1, Bio Tek, United States) were taken 0 and 24 h after shikonin addition to record and calculate the scratch area.

Migration rate (%) = [(wound area_0h_-wound area_24h_)/wound area_0h_] x100%. When using ImageJ for analysis, all the parameter settings of the images were kept consistent to minimize the errors caused during the data analysis process. ImageJ was used to analyze and set three independent results.

### 2.6 RNA extraction

Cells were seeded in 6-well plates at a density of 3 × 10^5^ cells per well. After 24 h of incubation, shikonin was added (MDA-MB-231: 0, 0.484 μM, MDA-MB-468: 0, 1.070 μM). The cells were incubated for another 24 h. Then, the medium was discarded, and the cells were washed once with pre-cooled PBS. Next, 1 mL of Total RNA Extractor (Trizol) reagent (Sangon Biotech, B511311, Shanghai, China) was added to each well, and the lysate was collected into a sterile, RNase-free 1.5 mL Eppendorf tube. Total RNA was extracted according to the reagent instructions.

### 2.7 mRNA high-throughput sequencing

RNA concentration and quality were measured using a Nanodrop 2000 (Thermo Scientific). Paired-end sequencing was performed using the Illumina HiSeq2500. RNA-seq analysis was conducted using Tophat2 (http://ccb.jhu.edu/software/tophat) in comparison to the human reference genome hg38. Transcript read counts were calculated using featureCounts (http://subread.sourceforge.net). Differential expression gene analysis (fold change >1.5, P < 0.05) was performed using DESeq2 (version 3.12) in RStudio 4.0. Heatmap visualization was performed using with Pearson correlation coefficient as the distance metric for different expressed genes (DEGs).

### 2.8 RT-qPCR

After treating with shikonin for 24 h, cells in the six-well plate were collected and washed once with PBS pre-cooled at 4 °C. Next, 1 mL of TRIzol was added to each well. Total RNA was extracted according to the manufacturer’s instructions, using sterile and enzyme-free consumables throughout the process. The RNA concentration was measured, and reverse transcription was performed using a reverse transcription kit (TaKaRa Biotech, RR047A, Beijing, China) to synthesize cDNA. Subsequently, cDNA was used as a template for fluorescence quantification using FastStart Essential DNA Green Master Mix (Roche, 06924204001, Shanghai, China). The mRNA expression was analyzed using the 2^−ΔΔCT^ method during data processing. The primers used for RT-qPCR are listed in Table 2.

**TABLE 2:** The information of the gene primers.

Gene	Forward primer	Reverse primer
*GAPDH*	5′-TGA​GTA​CGT​CGT​GGA​GTC-3′	5′-GGA​GGC​ATT​GCT​GAT​GAT​C-3′
*BIRC3*	5′-GTT​CAT​CCG​TCA​AGT​TCA​AGC-3′	5′-GGC​AGC​ATT​AAT​CAC​AGG​AGT-3′
*GIL3*	5′-CAT​AGT​GAA​GTG​CTC​CAC​TC-3′	5′-CAC​TCG​ATG​TTG​AAG​GTT​CC-3′
*TPR*	5′-CAG​AGG​ATG​TTA​AAC​GTC-4′	5′-CAG​CCA​TGT​ATT​CTG​ACT-4′
*MTOR*	5′-TCC​GAG​AGA​TGA​GTC​AAG​AGG-5′	5′-CAC​CTT​CCA​CTC​CTA​TGA​GGC-5′
*CCND1*	5′-TGA​ACT​ACC​TGG​ACC​GCT​TC-6′	5′-TTG​TTC​ACC​AGG​AGC​AGC​T-6′
*E2F3*	5′-ACA​AGG​CAG​CAG​AAG​TGC-7′	5′-GAC​TGA​GCT​CGG​TCA​CTT-7′
*CDK6*	5′-GTC​GAT​CAA​GAC​TTG​ACC​AC-8′	5′-CTG​GTC​ACC​AGA​ATG​TTC​TG-8′

### 2.9 Molecular docking

Molecular docking and dynamic simulation analyses were conducted using BIOVIA Discovery Studio 2021 with the CHARM force field. The structure of shikonin was obtained from the PubChem database and prepared using the “Prepare Ligands” module, obtaining 10 conformations. For docking, the structures of the mTOR kinase domain with X6K (PDB code: 4JT6) and the FRB domain with rapamycin (PDB code: 4DRJ) were used. The proteins were adjusted using the “Protein Preparation” module, including the addition of hydrogens, the removal of water molecules, and the optimization of energy. Ligand-binding sites were defined based on the original ligand (X6K or rapamycin). CDOCKER energy was used to evaluate the binding affinities of the different conformations. The spatial structures and binding sites were analyzed using Discovery Studio and PyMOL 3.7.

To further validate the docking results, 200 ps molecular dynamics (MD) simulation was performed. After solvation and two-step minimization, the system was heated from 50 K to 300 K. Equilibration was done for a simulation time of 20 ps. Then, 200 ps MD simulations were being run under the NPT ensemble at 300 K. Timestep of 2 fs and CHARMM36 m force field were applied. The stabilities of the complexes were assessed by root mean square deviation (RMSD) and root mean squared fluctuation (RMSF) time profiles.

### 2.10 Statistical analysis

All experiments were performed in triplicate (n = 3), and the results are reported as the mean ± SEM. Statistical analyses were conducted using GraphPad Prism 10.0 (GraphPad Software, San Diego, CA, United States). For comparisons between two groups, Student’s t-test was employed (data were tested for normality and equal variance before applying Student’s t-test), also with a p-value <0.05 indicating statistical significance.

## 3 Results

### 3.1 Shikonin inhibits the proliferation, growth, and migration of TNBC cell lines MDA-MB-231 and MDA-MB-468

**FIGURE 1:**
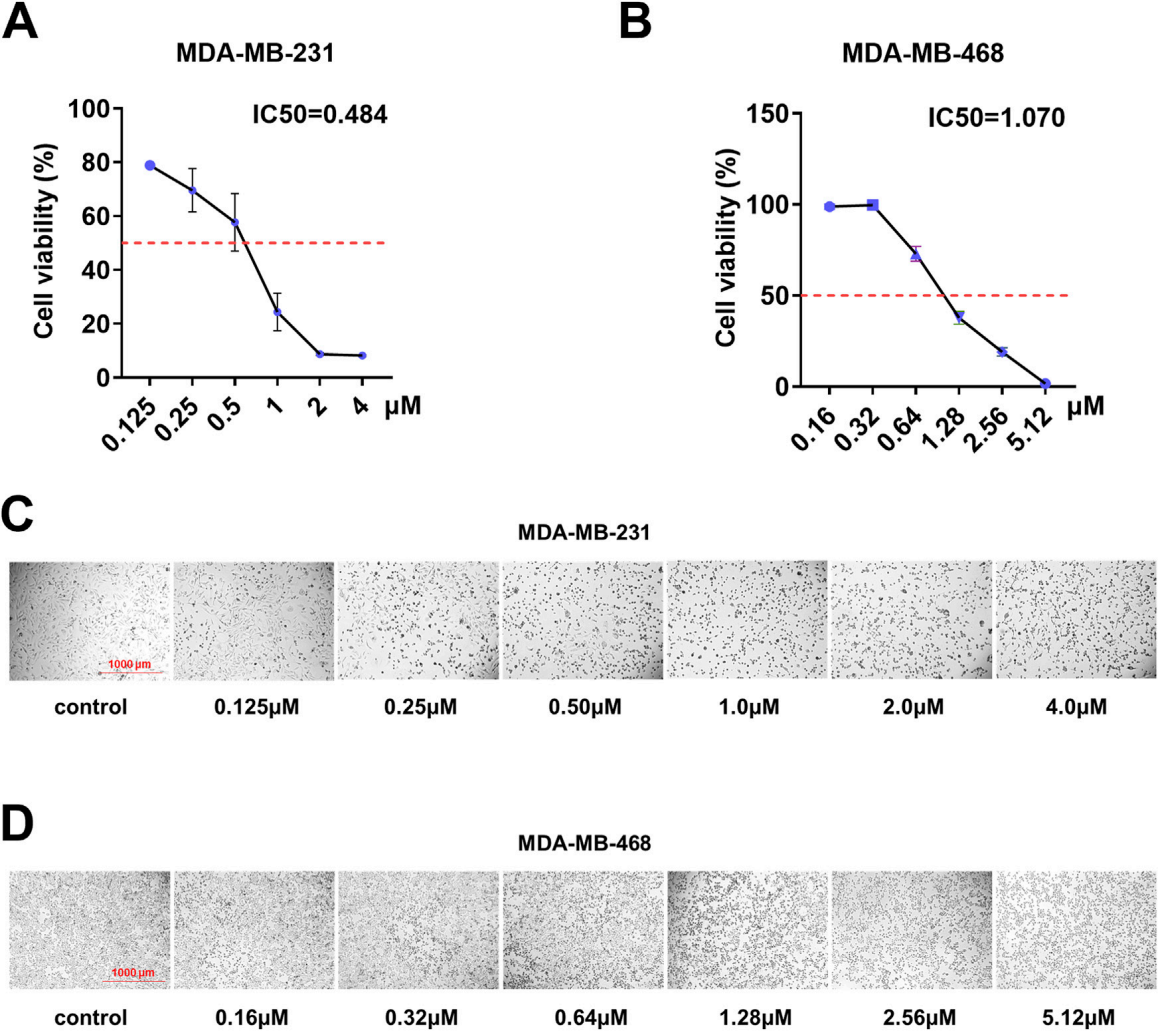
Shikonin inhibited the cell viability of MDA-MB-231 and MDA-MB-468 cells in a dose-dependent manner. **(A,B)** IC_50_ of shikonin inhibition in breast cancer cells. **(C,D)** MDA-MB-231 and MDA-MB-468 cell numbers and morphological changes were positively correlated with shikonin concentrations.

To investigate the inhibitory effects of shikonin on the TNBC cell lines MDA-MB-231 and MDA-MB-468, we conducted cellular experiments for verification. Initially, MDA-MB-231 and MDA-MB-468 cells were treated with different concentrations of shikonin. Cell viability was assessed using a CCK8 kit, revealing IC_50_ values of 0.484 μM for MDA-MB-231 and 1.070 μM for MDA-MB-468 (Figures 1A,B). Notably, as the concentration of shikonin increased, cell viability decreased (Figures 1C,D), indicating a concentration-dependent inhibitory effect of shikonin on TNBC cell viability.

**FIGURE 2:**
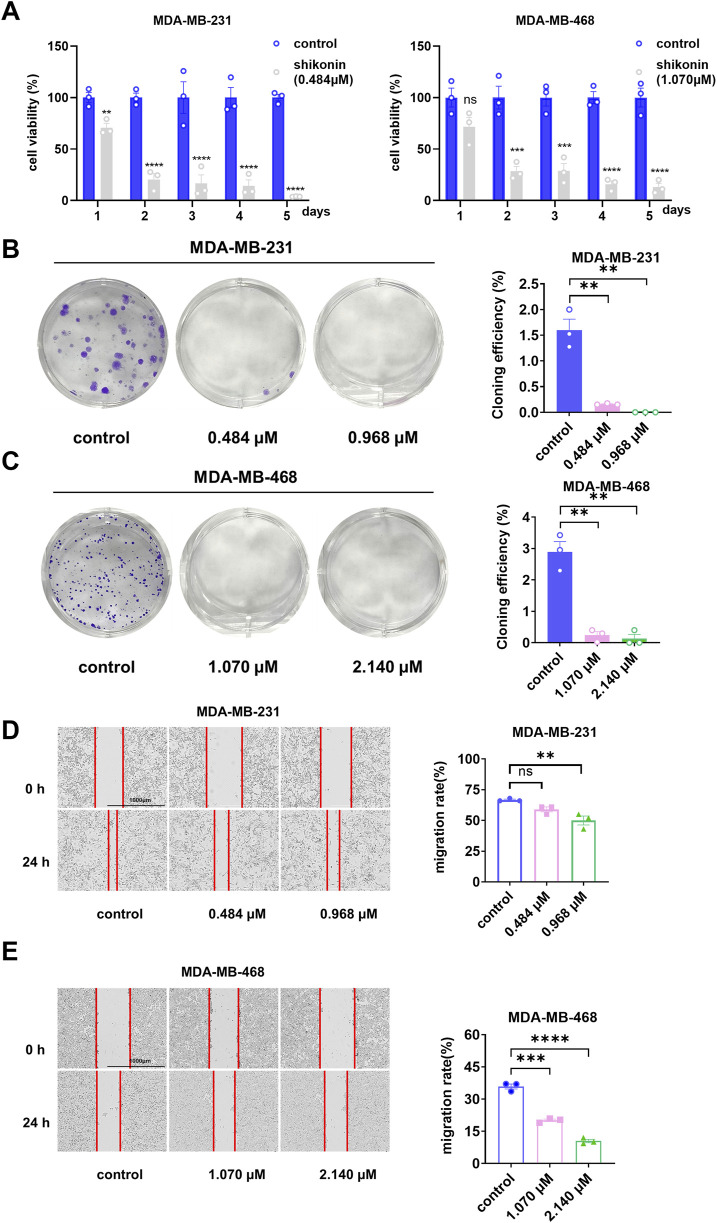
Shikonin inhibited the proliferation and migration abilities of MDA-MB-231 and MDA-MB-468 cells in a time-dependent manner. **(A)** The cell viability of shikonin was measured by CCK8 at 24 h, 48h, 72h, 96h and 120h, respectively. The viability of MDA-MB-231 and MDA-MB-468 cells decreased in a time-dependent manner with shikonin. **(B,C)** In the colony formation assay, 0.484 μM and 0.968 μM shikonin acted on MDA-MB-231 cells, while 1.070μM and 2.140 μM shikonin acted on MDA-MB-468 cells significantly reduced the number of clones **(D,E)**. Shikonin significantly inhibited the migratory viability of MDA-MB-231 and MDA-MB-468 cells at 24 h. The cell migration pictures were gained by 200 times magnification under the microscope. **** represents p < 0.0001, *** represents p < 0.001, ** represents p < 0.01.

Based on these findings, we conducted cell proliferation, colony formation, and wound-healing assays to examine the effects of shikonin on the proliferation, growth, and migration of human breast cancer cell lines MDA-MB-231 and MDA-MB-468. According to the results of the cell proliferation experiments (Figures 2A,B), we observed a significant decrease in the viability of MDA-MB-231 and MDA-MB-468 cells following treatment with shikonin, and this trend became more pronounced over time. Furthermore, the results of the clonogenic assay (Figures 2C,D) revealed that for MDA-MB-231 cells, the colony formation rate in the normal group was 1.6%, while in the treatment group with 0.484 μM shikonin, it decreased to 0.158%, and complete inhibition of colony formation was observed in the 0.968 μM shikonin group. Similarly, for MDA-MB-468 cells, the colony formation rate in the normal group was 2.89%, which was reduced to 0.241% upon treatment with 1.070 μM shikonin, and further decreased to 0.133% in the 2.140 μM shikonin group. These findings strongly demonstrate that shikonin exerts a significant inhibitory effect on the proliferation of the human breast cancer cell lines MDA-MB-231 and MDA-MB-468.

To further investigate whether shikonin affected the migration ability of MDA-MB-231 and MDA-MB-468 cells, we performed wound-healing assays (Figures 2E,F). The results showed that the migration rate of the normal MDA-MB-231 cell group was 66.7%, which decreased to 59% in the treatment group with 0.484 μM shikonin, and further reduced to 50% in the 0.968 μM shikonin group. For MDA-MB-468 cells, the migration rate of the normal group was 35.9%, which dropped to 20.9% upon treatment with 1.070 μM shikonin, and was only 10.5% in the 2.140 μM shikonin group. These data indicate that shikonin effectively inhibits the migration of the human breast cancer cell lines MDA-MB-231 and MDA-MB-468.

### 3.2 Systematic bioinformatics analysis identified oncogenes regulated by shikonin in TNBC

**FIGURE 3:**
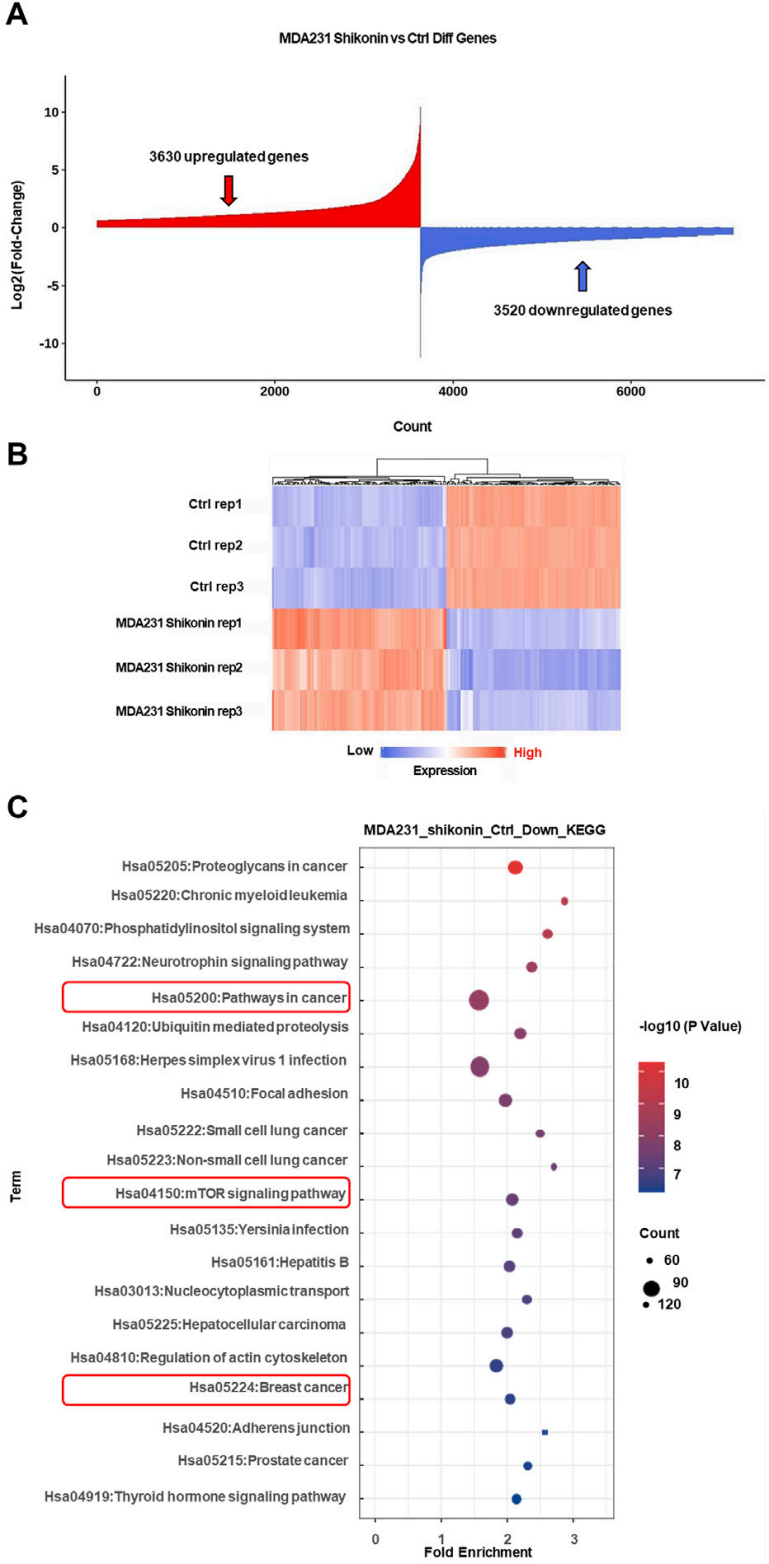
High through-put sequencing analysis revealed mTOR signaling pathway may be critical for TNBC progression after shikonin treatment. **(A)** A total of 3,630 upregulated genes and 3,520 downregulated genes were obtained by mRNA sequencing analysis under the effect of shikonin. **(B)** Heat map of the top 100 differential genes after treatment by shikonin in MDA-MB-231 cells. **(C)** The downregulated genes are mainly involved in cancer pathways, mTOR signaling pathway and breast cancer by KEGG profiling.

To explore the molecular mechanisms underlying the inhibitory effects of shikonin on breast cancer cell proliferation, growth, and migration, high-throughput sequencing was performed. The results revealed that 48 oncogenes were downregulated upon shikonin treatment, including *EGFR*, *MTOR*, *NOTCH1*, and *STAT3* (Figures 3A,B). KEGG enrichment analysis further revealed that shikonin suppressed key tumorigenesis-related pathways, including the mTOR signaling pathway and pathways implicated in cancer and breast cancer (Figure 3C).

**FIGURE 4:**
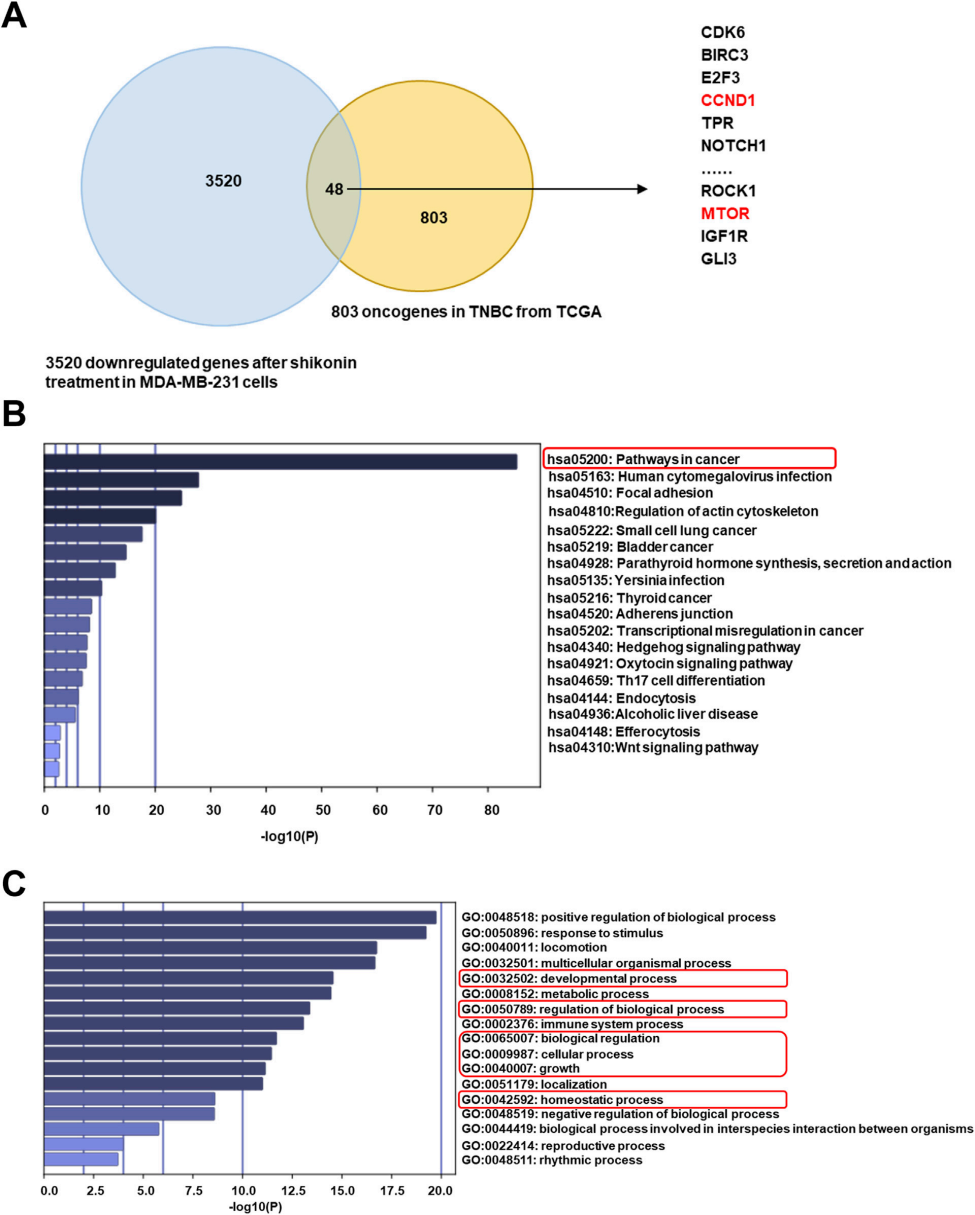
Shikonin may inhibit oncogenes through mTOR signaling pathway in breast cancer. **(A)** A total of 48 suppressed oncogenes among 3520 downregulated genes by shikonin. **(B)** KEGG profiling for the 48 downregulated oncogenes by shikonin. **(C)** GO analysis for the 48 downregulated oncogenes by shikonin.

Oncogenes can cause cancer when mutated or abnormally expressed. By targeting these oncogenes, shikonin exerts anticancer effects by inhibiting cell proliferation, inducing apoptosis, and suppressing tumor progression. Using the ONGene database, which includes 803 oncogenes annotated in human tumors, we cross-referenced the genes downregulated by shikonin with this list to identify potential oncogenes regulated by shikonin in TNBC. This analysis revealed 48 such genes (Figure 4A), including *CDK6*, *CCND1*, and *MTOR*. Subsequent KEGG and GO analyses of these 48 downregulated oncogenes revealed that the mTOR signaling pathway may be involved in the inhibition of the malignant progression of TNBC by shikonin (https://bioinfo-minzhao.org/ongene/) (Figures 4B,C). The PI3K/AKT/mTOR signaling pathway is one of the most frequently activated pathways in breast cancer, regulating the proliferation and growth of tumor cells (Miller et al., 2011). After integrating the results from the cellular experiments, high-throughput sequencing, and KEGG analysis, we hypothesized that alterations in the mTOR-related pathway are crucial for the regulatory effects of shikonin on breast cancer progression.

### 3.3 Shikonin inhibits *MTOR* expression and key genes in related signaling pathways

**FIGURE 5:**
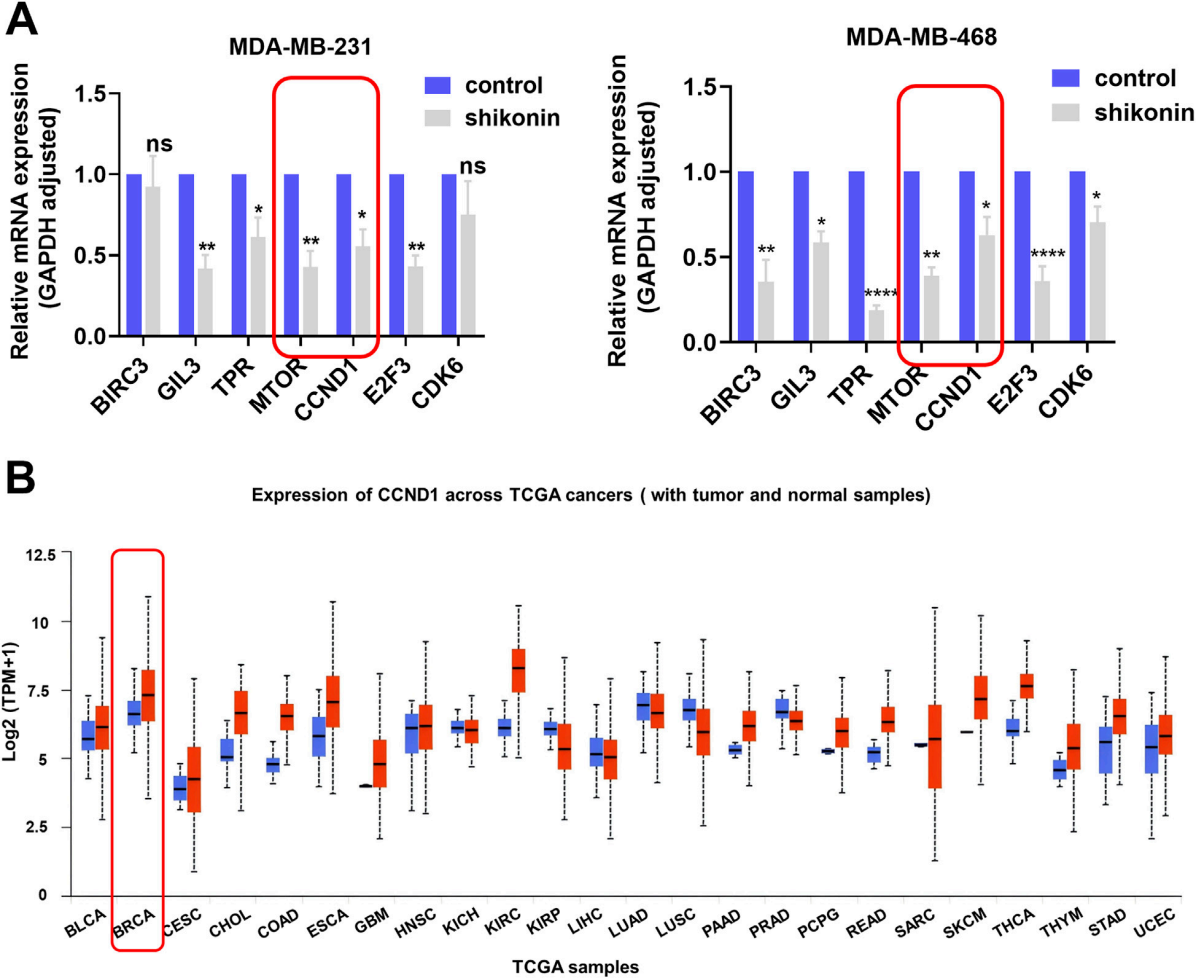
Shikonin decreased oncogenes *CCND1* in the mTOR pathway. **(A)** *BIRC3*, *GIL3*, *TPR*, *MTOR*, *CCND1*, *E2F3* and *CDK6* were suppressed in MDA-MB-231 and MDA-MB-468 cells after shikonin treatment as verified by qPCR. **(B)** Panoncogene expression profiles of *CCND1*. **** represents p < 0.0001, ** represents p < 0.01, * represents p < 0.05.

We performed RT-qPCR to assess the mRNA expression levels of oncogenes that were significantly downregulated following shikonin treatment (Figure 5A). Notably, the expression of *MTOR* and its downstream gene, *CCND1*, markedly decreased. Data analysis from TCGA (accessed through the UNLCAN online database) revealed that *CCND1* expression was significantly elevated in breast cancer cells compared with normal breast cells (Figure 5B). Consistently, another study demonstrated that mTOR pathway blockade can suppress the translation of a subset of mRNAs in the 5′-untranslated region that are crucial for cell cycle proliferation and the transition from the G1 phase to the S phase (Dutcher, 2004). These mRNAs encode proteins such as c-MYC and CCND1. CCND1 binds to CDK4/CDK6 to promote RB phosphorylation, thereby facilitating cell cycle progression and DNA replication. Inhibition of CCND1 expression impedes cell cycle progression. Therefore, we propose that shikonin downregulates *CCND1* expression by inhibiting the activity of the mTOR pathway, thereby suppressing the proliferation and growth of TNBC cells, and inhibiting the malignant progression of TNBC.

### 3.4 Molecular docking confirmed the interaction between shikonin and mTOR protein

**FIGURE 6:**
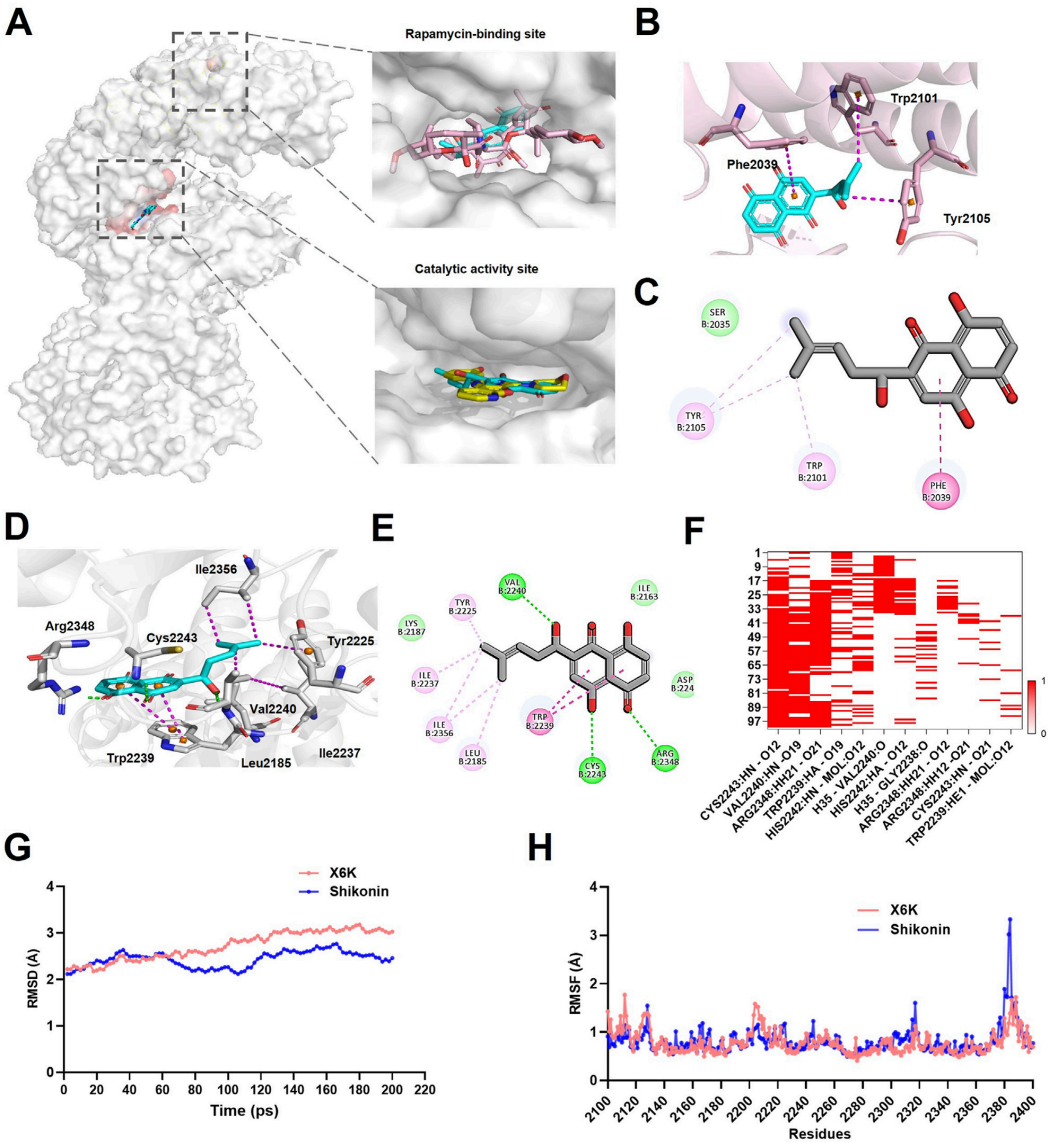
Shikonin could bind to mTOR. **(A)** Shikonin occupied the mTOR kinase domain with X6K and FRB domain with rapamycin. mTOR (grey), Shikonin (blue), rapamycin (pink), and X6K (yellow). **(B)** Interaction of Shikonin with mTOR at the rapamycin-binding site. **(C)** Diagram of Shikonin interactions in the rapamycin-binding site. **(D)** Interaction of Shikonin with mTOR at the catalytic activity site. **(E)** Diagram of Shikonin interactions in the catalytic activity site. The hydrogen bond was depicted in a green line, and the hydrophobic interaction was shown as a purple line. **(F)** The heatmap of hydrogen bond interactions. **(G)** RMSD analyses the stability of mTOR-shikonin and mTOR-X6K complexes during a 200-ps MD simulations. **(H)** RMSF fluctuations of mTOR-shikonin and mTOR-X6K complexes over amino acid residues. RMSD: root mean square deviation; RMSF: root mean square fluctuation.

To further determine whether shikonin interacts with mTOR, we used molecular docking to analyze its affinity. Studies have shown that mTOR function is regulated by the catalytic activity and allosteric sites of rapamycin (Stary et al., 2022; Yang H. et al., 2013). Therefore, shikonin was docked with different mTOR protein structures to explore its affinity and specificity. Our results showed that shikonin could bind to both the catalytic and rapamycin sites (Figure 6A). At the rapamycin-binding site, shikonin primarily interacted through hydrophobic interactions with Tryptophan (Trp) 2101 and Tyrosine (Tyr) 2105, along with π–π stacking interactions with Phenylalanine (Phe) 2039. The hydrophobic interactions with Trp 2101 and Tyr 2105 were all within 5.0 Å (Figures 6B,C), suggesting stable binding between shikonin and mTOR.

In the case of the catalytic activity site, the residues analysis demonstrated that shikonin formed hydrogen bonds with Val 2240, Cys 2243, and Arg 2348, with hydrogen bond distances of 2.2, 2.0, and 2.3 Å, respectively (Figures 6D,E). A strong hydrophobic interaction was also formed between the naphthalene ring of shikonin and Tyr2239 through π–π stacking. Additionally, shikonin adopted hydrophobic interactions with Leu2185, Try2225, Ile2237, and Isoleucine (Ile)2356 (Figures 6D,E), which further enhanced the stability of the shikonin-binding mode.

Molecular dynamics simulations were performed to further validate the stability and binding patterns of mTOR-shikonin. Specifically, we investigated the stability of the mTOR-shikonin complex in the kinase domain, a crucial active site for mTOR. The heat map of the hydrogen bond interactions revealed that shikonin consistently formed hydrogen bonds with key mTOR residues, including Valine (Val) 2240, Cysteine (Cys) 2243, and Arginine (Arg) 2348 (Figure 6F), suggesting these bonds were durable and stable. The RMSD of the mTOR-shikonin and mTOR-X6K complexes fluctuated by 2.1144–2.7644 and 2.2198–3.0255 Å respectively, after 200 ps of simulation time (Figure 6G). The average the root mean square deviation (RMSD) value of the mTOR-shikonin complex was 2.4299 Å, which was slightly lower than the average value of 2.7227 Å for the mTOR-X6K complex, indicating that both complexes were in a stable state. Furthermore, root mean square fluctuation (RMSF) values for core residues in both complexes remained under 1 Å, especially for Val 2240, Cys 2243, and Arg 2348 (Figure 6H). Taken together, these results suggest that shikonin has high binding affinity for mTOR.

## 4 Discussion

Breast cancer is the most commonly diagnosed malignancy worldwide, with an estimated 2.3 million new cases in 2020. In women, breast cancer accounts for 25% of cancer cases and 16% of cancer-related deaths (Sung et al., 2021). TNBC accounts for 10%–15% of all breast cancers and is characterized by the absence of ER, PR, and HER2 receptor expression. TNBC has the worst prognosis of any breast cancer type because of poor chemotherapy response, lower survival, and higher recurrence rate. Chemotherapy, radiotherapy, targeted therapy, and immunotherapy are the main methods currently employed for treating TNBC (Derakhshan and Reis-Filho, 2022). However, they still face great challenges in terms of adverse drug reactions, multidrug resistance, and treatment effectiveness, necessitating the development of alternative treatment options (Iranzadeh et al., 2024). Traditional Chinese medicine and natural medicine offer distinct advantages, such as multi-target effects and minimal side effects, making them promising complementary and alternative therapies in breast cancer treatment (Yang et al., 2021). In particular, Chinese herbal medicine-derived phytochemicals, such as shikonin, have demonstrated significant antitumor effects in various types of cancer (Liang et al., 2024). Our prior studies have shown shikonin’s inhibitory effects on lung and colon cancers, along with its underlying molecular mechanisms (Liu et al., 2024; Zhao et al., 2024). In addition, it has also been reported that shikonin exerts anti-cancer activity in thyroid cancer, non-small cell cancer, bladder cancer and other cancers (Li et al., 2017; Yang Q. et al., 2013; Wang et al., 2018). Among these reports, there are some that focus on the anti-cancer mechanism of TNBC. For example, some researchers have discovered that shikonin inhibits the progression of TNBC by suppressing *IMPDH2* (Wang et al., 2021). It is well known that mutations in proto-oncogenes are key to tumor formation (Martinez-Jimenez et al., 2020). However, it remains unclear whether shikonin exerts its antitumor effects by suppressing oncogenes in breast cancer.

This study provides compelling evidence that shikonin exerts antitumor activity in breast cancer cell lines by targeting the mTOR signaling pathway. Our mRNA-seq analysis revealed significant downregulation of 3,505 genes in shikonin-treated MDA231 cells, with 37 oncogenes in the mTOR pathway being notably suppressed. These findings suggest that shikonin exerts its anticancer effects by inhibiting the mTOR signaling pathway, which is frequently activated in various cancers, including breast cancer. The downregulation of *MTOR* and its downstream gene, *CCND1*, was further confirmed via RT-qPCR, thereby strengthening our hypothesis that shikonin targets the mTOR pathway (Glaviano et al., 2023). The molecular docking study also provided valuable insights into the interaction between shikonin and mTOR protein, revealing that shikonin primarily interacts with mTOR through hydrophobic bonds and π–π stacking.

Despite these promising results, our study has several limitations. First, our findings are based on *in vitro* experiments using a single breast cancer cell line. To fully elucidate the anticancer effects of shikonin, further studies are required to investigate its activity in other cancer cell lines and animal models. In addition, the mechanisms underlying the inhibition of the mTOR pathway by shikonin remain unclear. Future studies should explore the upstream and downstream signaling events involved in shikonin-induced mTOR inhibition.

## 5 Conclusion

**FIGURE 7:**
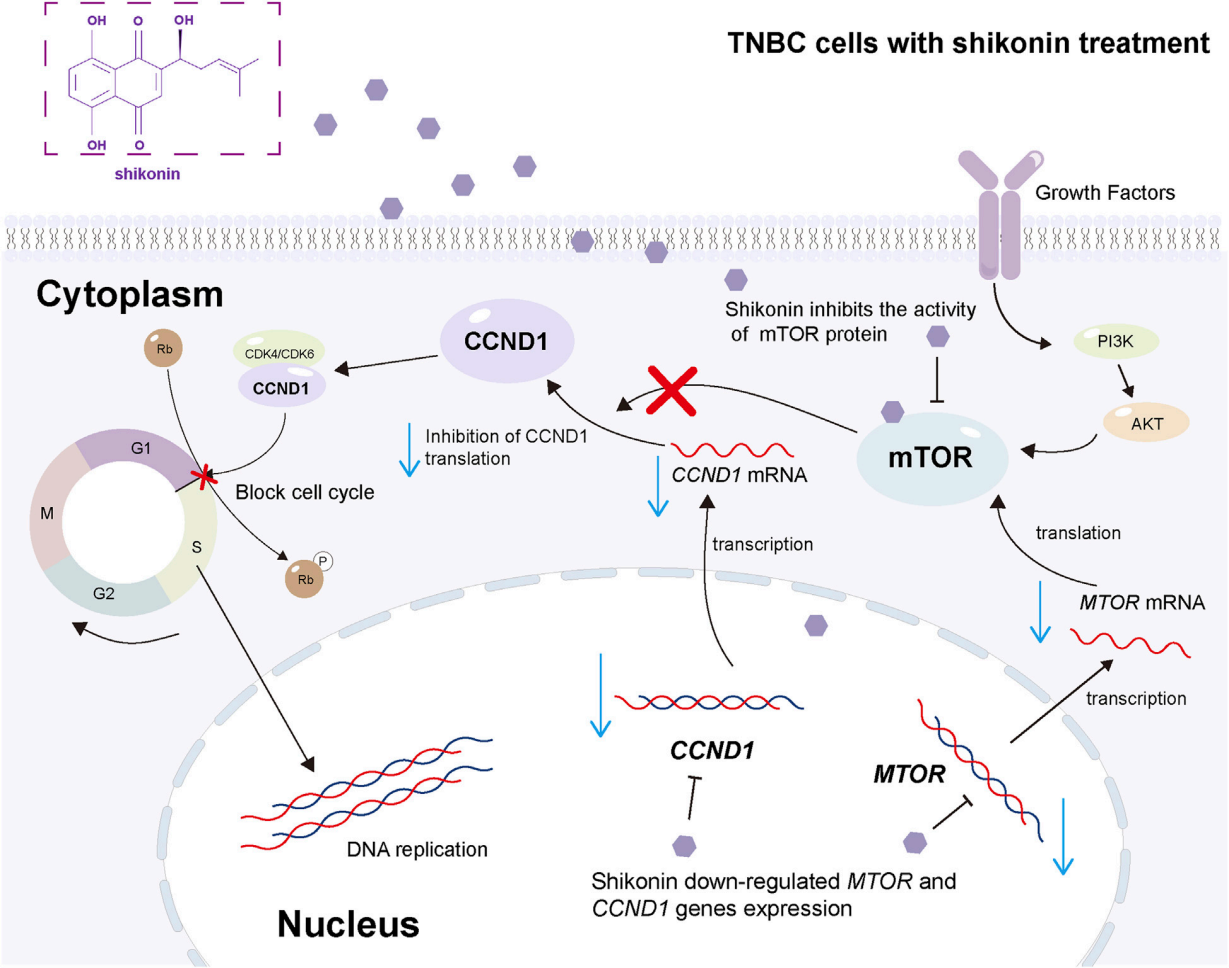
The schematic diagram of the mechanism for the present work. Shikonin acts both in the nucleus and cytoplasm to downregulate specific genes and inhibit the mTOR pathway, thereby inhibiting the malignant progression of TNBC. In detail, shikonin downregulates the expression of *MTOR* and *CCND1* genes in the nucleus; meanwhile, shikonin downregulates the mRNA expression of *MTOR* and *CCND1*, inhibits the activity of mTOR protein through hydrophobic bond and π–π bond binding, thereby inhibiting the activity of mTOR pathway, blocking the cell cycle, and inhibiting the malignant progression of TNBC in the cytoplasm.

Our study revealed the underlying mechanism of action of shikonin in the treatment of breast cancer. As a natural bioactive compound, shikonin specifically inhibits the overactivation of the mTOR signaling pathway and effectively downregulates the expression of its key downstream effector gene, *CCND1*. Reduced expression of *CCND1*, a critical regulator of the cell cycle, leads to a significant decrease in breast cancer cell proliferation (Figure 7). This series of actions collectively forms the molecular basis for the anti-breast cancer effects of shikonin, offering new strategies and targets for breast cancer treatment. In summary, shikonin and its derivatives hold promise as potential drug candidates for inhibiting breast cancer progression and improving patient prognosis, meriting further investigation and development.

## Data Availability

The datasets presented in this study can be found in the NCBI repository, accession number GSE281168. Further inquiries can be directed to the corresponding authors.

## Abbreviations

TNBC, Triple-negative breast cancer; CCND1, Cyclin D1; mTOR, mammalian target of rapamycin; CDK6, Cyclin-Dependent Kinase 6; TCM, Traditional Chinese medicine; HER2, Human Epidermal Growth Factor Receptor 2; ER, expression of estrogen receptor; PR, progesterone receptor; EMT, epithelial-mesenchymal transition; DMSO, Dimethyl sulfoxide; PBS, Phosphate Buffered Saline; DMEM, Dulbecco’s Modified Eagle’s Medium; RPMI, Roswell Park Memorial Institute; CCK8, Cell Counting Kit-8; RNA-seq, RNA sequencing; RT-qPCR, Reverse Transcription Quantitative Real-time polymerase chain reaction; TCGA, The Cancer Genome Atlas; Trp, Tryptophan; Tyr, Tyrosine; Phe, Phenylalanine; Ile, Isoleucine; Val, Valine; Cys, Cysteine; Arg, Arginine; RMSD, root mean square deviation; RMSF, root mean square fluctuation; siTGF-β, tumor growth factor β small interfering RNA.
